# A shotgun-induced nonunion humeral fracture treated by Masquelet technique and arthrodesis: a case report

**DOI:** 10.1097/MS9.0000000000000457

**Published:** 2023-07-28

**Authors:** Alireza Taabbodi, Parmida Shahbazi, Payman Mohammad Hosseini Azar, Niloofar Gholami, Dorsa Hadavi, Arvin Najafi

**Affiliations:** a Orthopedic Surgery Ward, Shahid Madani Hospital, Alborz University of Medical Sciences, Karaj; b Cardiovascular Research Center, Alborz University of Medical Sciences, Alborz, Iran; c Department of Radiology, Imam Khomeini Hospital Complex, Tehran University of Medical Sciences, Tehran, Iran

**Keywords:** Case reports, Firearms, humerus, Masquelet technique, upper extremity

## Abstract

**Introduction and importance:**

The Masquelet technique remains one of the procedures with low rates of failure and infection. The use of this technique in humeral defects is still rare.

**Case presentation:**

A 38-year-old male patient with an open humeral comminuted fracture induced by shotgun injury was referred to our hospital. The Masquelet technique was chosen as the best option with a lower risk of infection and the lower expenses at this stage due to the second time of open reduction and internal fixation and bone graft failure, low patient compliance, and the increasing size of the defect due to bone absorption. An arthrodesis procedure was performed 5 days after the second Masquelet stage as restoring the elbow joint’s range of motion was impossible.

**Clinical discussion:**

The Masquelet technique, is a two-step surgical procedure to manage pseudoarthroses and bone defects. Various surgical options are available for performing this procedure. There are several reasons behind the rising popularity of this technique during recent years. Some of these reasons include the reproducibility of this technique, as well as requiring less time, not being technically challenging, and having fewer neurovascular complications.

**Conclusion:**

This case was one of the limited examples of successful implementation of the Masquelete procedure on severe traumatic injuries of the upper limb with bone defects providing more evidence on the safety and efficacy of this technique in similar conditions.

## Introduction

Highlights

- The Masquelet technique has been found to be an effective and safe treatment option for humeral fracture with nonunion caused by a shotgun injury.
- The use of Masquelet may be a cost-effective approach for managing nonunion in humeral fractures.
- There is potential for Masquelet to be effective in treating nonunion in both upper and lower limb fractures.

Nearly 4% of the humeral shaft fractures with plate fixation result in nonunion^1^. Segmental bone defects following traumatic injuries are among the most complex and challenging clinical situations causing significant longstanding morbidities^2^. Induced membrane technique (IMT), also known as the Masquelet technique since 2000^3^, consists of two surgical steps with the interval of 4–8 months used for the treatment of nonunion fractures or bony defects. The first surgery is performed to create a pseudo-synovial membrane providing vascular supply in bony defect through debridement of damaged tissue, placement of a cement spacer in the defect space, and immobilization with an external fixator. The cement spacer is then removed in the second surgery, and the defect space is filled with autologous bone graft, getting immobilized afterward^4,5^.

Until now, this procedure has been mostly performed on the lower extremity defects. Despite many studies confirming the efficiency of the Masquelet technique on the upper extremity, its use in humeral defects is still scarce^6,7^.

This paper reports the use of the Masquelet technique for the reconstruction of a nonunion humeral fracture caused by a shotgun injury.

## Presentation of case

**Figure 1:**
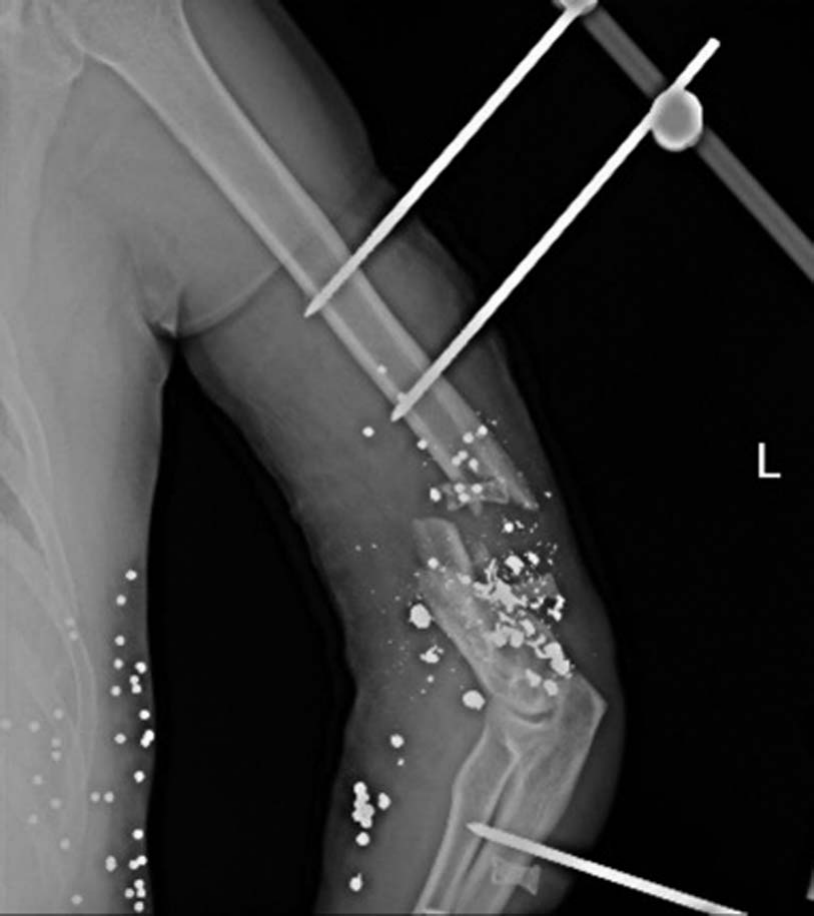
The initial X-ray at admission time, 13A3 fracture fixed with an external fixator in another hospital.

A 38-year-old man who worked as a labourer was brought to the emergency department of a tertiary referral trauma centre from the prison with an ambulance in November 2019. He complained of pain in his left arm with a history of the distal humeral comminuted open fracture followed by a shotgun 3 weeks before hospitalization with a 2 cm bone defect in length. The patient was treated in another hospital immediately after the injury by brachial artery grafting and reconstruction, an external fixator placement, and a long arm immobilizer splint [Figure 1]. A physical examination revealed a high radial nerve palsy, an impaired extension of the thumb and the wrist (wrist drop), and impaired sensation on the first webspace. The capillary filling and the distal pulse were intact. There was no mention of any history of drug use or familial drug use relevant to this case.

**Figure 2:**
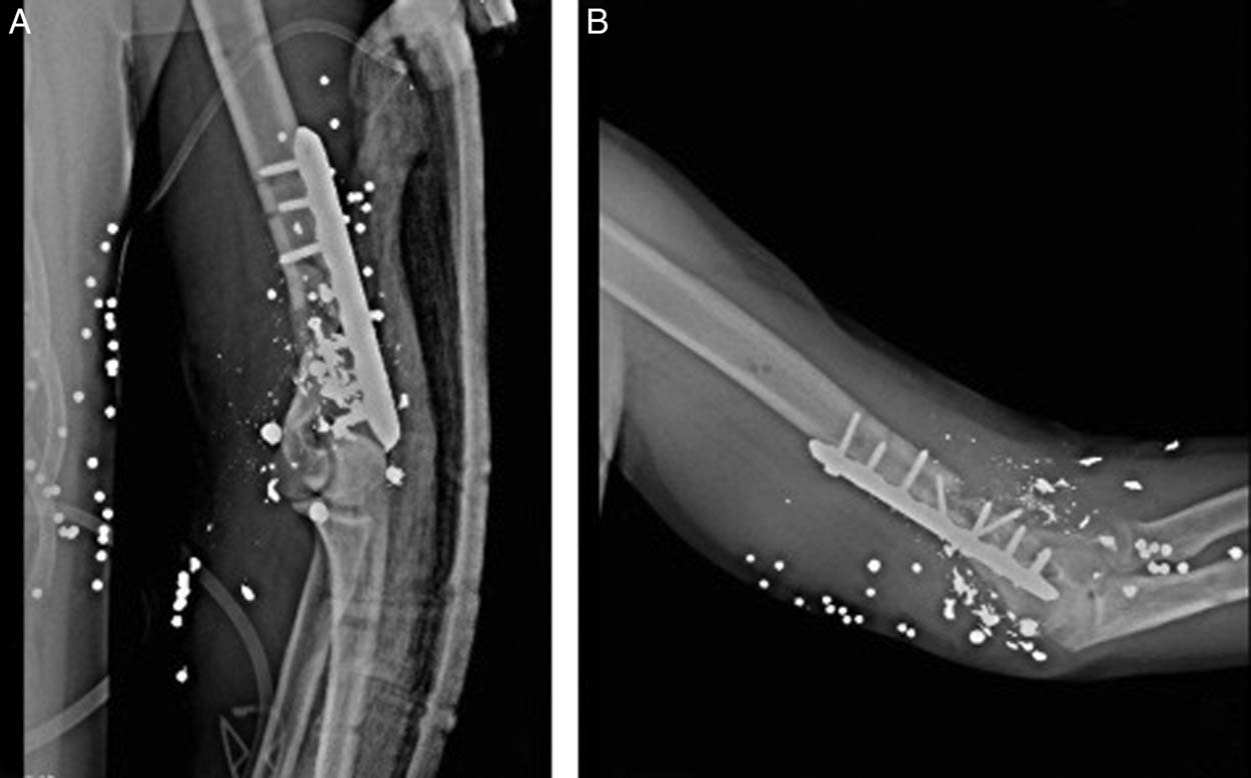
(A) Postoperative X-ray, after open reduction and internal fixation with allograft and splint fixation. (B) It is failure 6 months later.

He underwent surgery for the removal of external fixators. Subsequently, through dorsal triceps splitting, open reduction and internal fixation (ORIF) of the humeral shaft was performed with 4.5 mm, eight-hole, broad lateral distal humerus synthetic locking compression plate (LCP) produced by BoneTech company, Iran. Afterward, the lyophilized mineral bone allograft chips, an Iranian Tissue product, were placed in the defect space, and a long arm splint immobilized the limb [Figure 2A].

Six months later, he underwent another surgery with the impression of an aseptic nonunion fracture of the distal humeral metaphysis [Figure 2B].

**Figure 3:**
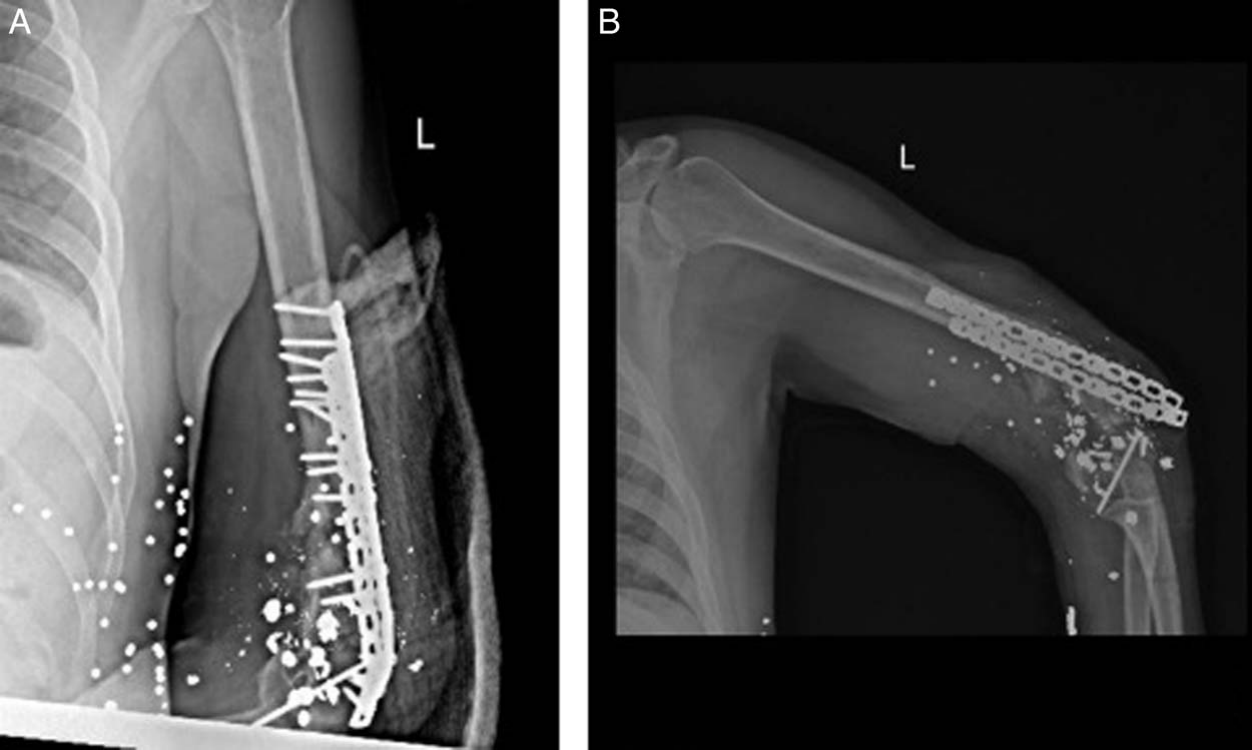
(A) The second fixation surgery in our hospital using the same approach as the previous fixation procedure. (B) plate failure in 2 and a half months later.

Through the previous postoperative scar incision, the ulnar nerve was identified and protected. There was no sign of purulent discharge or infection. The LCP was removed from the left humeral shaft then all the previous steps were precisely repeated with two 3.5 mm, 14-hole and 12-hole, BoneTech LCPs [Figure 3A].

Two and a half months later, on the third admission, the patient was referred to the hospital with a complaint of elbow pain and plate failure [Figure 3B]. The Masquelet technique was chosen as the best option at this stage due to the second time of ORIF and bone graft failure, low patient compliance, and the fact that the bone resorption process had increased the 2 cm defect to 4 cm.

**Figure 4:**
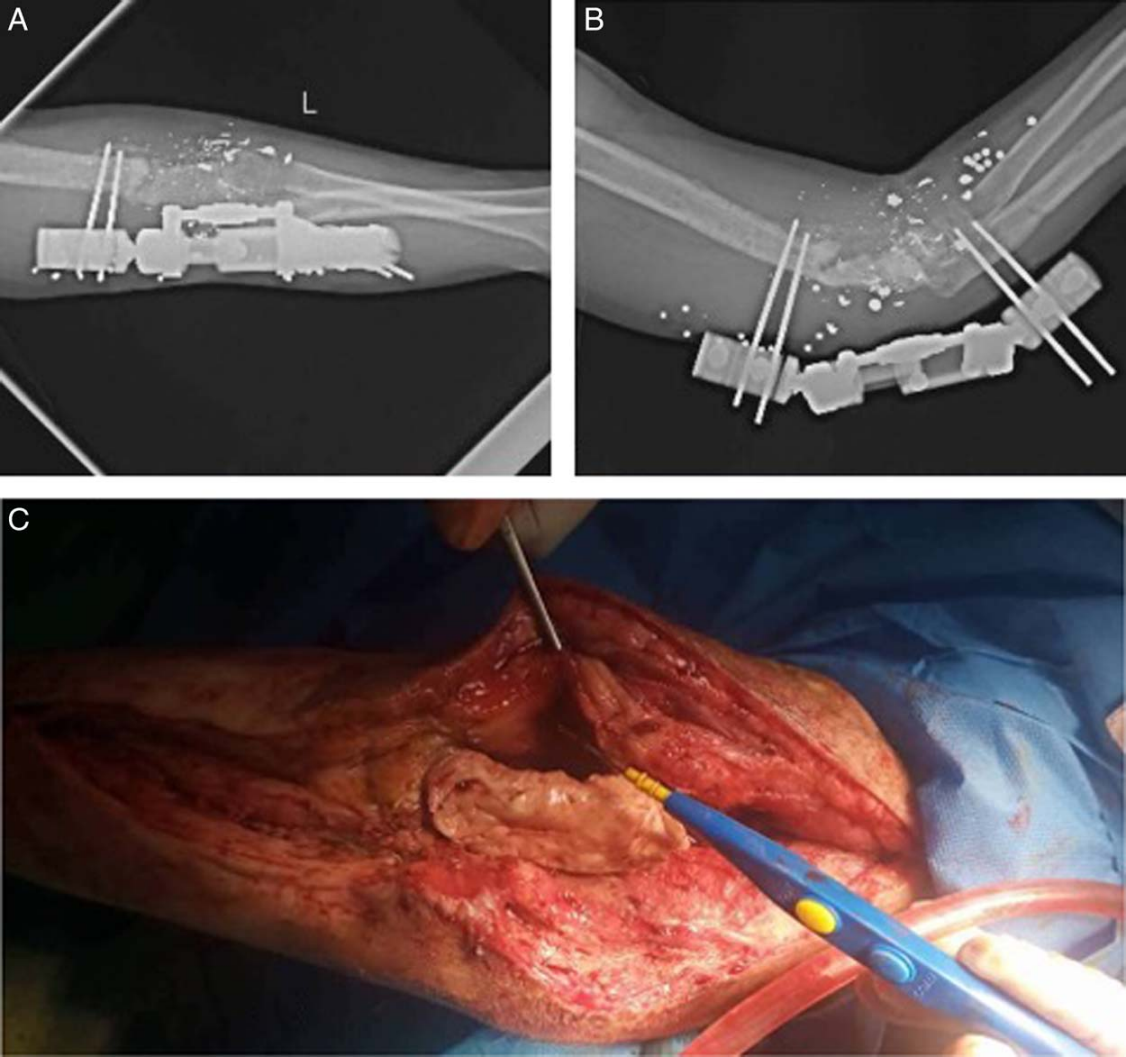
Bone cement (C) and external !xation (A and B), first step of the Masquelet technique.

The previous plates were removed during this operation. There was no sign of infection, and the cultures came back negative afterward. The antibiotic-impregnated cement spacer (CEMEX) was placed in the defect. It was stabilized with an external fixator (Dynamic Multiaxial Fixator, BEHTA) and a long arm splint. [Figure 4]

Two months later, the external fixator and the splint were removed. The cement spacer was also taken out 3 weeks later during the last surgery, and it was replaced with an autologous bone graft from the patient’s left iliac crest.

As a result of the previous bony absorption in the joint, restoring the elbow joint’s range of motion was impossible. Therefore, an arthrodesis procedure was planned 5 days after the second Masquelet stage with a BoneTech 14-hole, 4.5 mm, narrow limited-contact locking compression plate.

**Figure 5:**
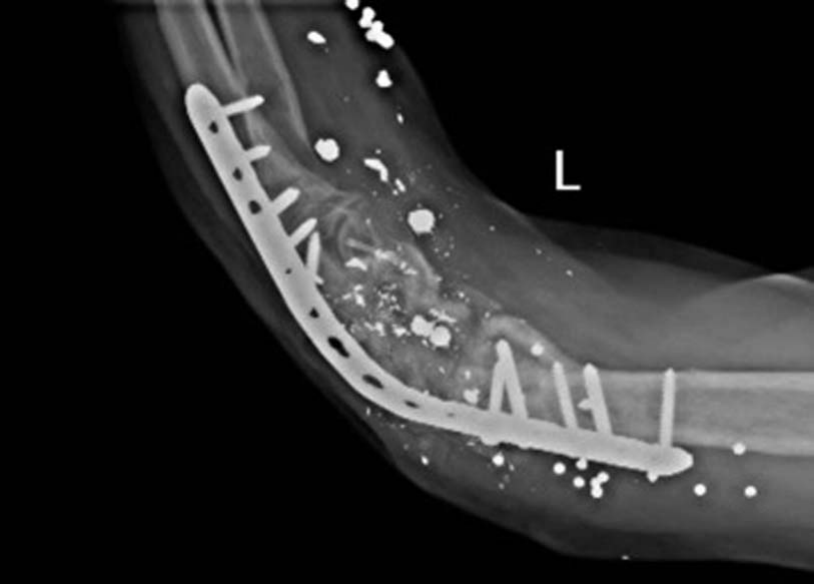
Two months later after arthrodesis and bone graft and before a tendon transfer.

Two months later, the radiographic images showed signs of union [Figure 5], and tendon transfer was carried out to repair the radial nerve palsy.

To provide additional information, it should be noted that an orthopaedist performed all the procedures in this case. Moreover, the tendon transfer procedure was carried out by an orthopaedist with a hand fellowship, indicating specialized training in hand surgery.

This paper has been reported in line with the SCARE criteria^8^.

## Follow-up

At the end of each hospitalization, the patient was discharged with oral antibiotics and recommended to visit the orthopaedic clinic a week later each month. Radiological and physical examinations were carried out on each follow-up visit.

The patient was followed for 6 months, and the first union was seen two months after the second Masquelet stage in the X-ray images. Furthermore, the patient was capable of actively extending his left wrist and fingers as well as fisting his hand after 3 months of the wide-awake local anaesthesia no tourniquet surgery^9^.

## Discussion

Induced Membrane Technique (IMT) is a two-step surgical procedure to manage pseudoarthroses and bone defects. Various surgical options are available for performing this procedure, including antibiotic-coated spacers, internal fixation devices, Reamer-Irrigator-Aspirator technique, iliac crest grafting, bone substitutes, and using growth factors^4^.

Amputation has classically been the procedure of choice for managing segmental long bone defects due to the technical difficulties of the alternative surgeries. On the other hand, Limb salvage techniques have just been developed during the last 50 years. For instance, performing massive cancellous bone autograft during the second world war was the major form of treatment^3,9^. There are several technical difficulties associated with the traditional bone graft techniques and other alternatives for managing segmental bone defects, such as uncontrollable graft resorption even in the presence of a well-vascularized recipient site^10^. As a more recent and more efficient therapeutic strategy, using an antibiotic cement spacer and placing the graft inside this space to provide an induced biomembrane has been explained^11,12^.

Previous investigations have shown promising results for using this procedure to treat post-traumatic bone defects^2^.

There are several reasons behind the rising popularity of this technique during recent years. Some of these reasons include the reproducibility of this technique, as well as requiring less time, not being technically challenging, and having fewer neurovascular complications. However, there are a few contraindications for this procedure^12^. The patient’s compliance is less extensively required (compared to bone transport), and the fact that this technique is not length-dependent^13,14^.

The previous systematic review about IMT on long bones reported fracture union in 89.7% of the cases, and the infection was eradicated in 91.1%. It should be considered that although IMT is designed to achieve bone union and lower the risk of infection, some adverse complications such as the persistence of infection or nonunion have been reported in a few cases before^4,15^.

Based on the report of Masquelet et al^15^, the defect size is not a determinant factor, and the procedure is performed on a various range of defects (from 0.6 to 26 cm). Failure of one or both of the IMT steps happens in a minority of cases. Re-intervention and further surgeries are required in case of the persistence of infection or other complications^4^.

Considering that our patient came from a lower-income population without reliable insurance, a less expensive reconstruction method was favourable. Furthermore, due to the patient’s poor compliance, fewer follow-up visits, and the lower risk of infection in the Masquelet technique made it a favourable option in managing this particular case.

In another study, Apard *et al.* ^9^. reported promising results for the procedure in a series of 12 patients with 6 cm segmental defects in the tibia. In all of these subjects, an intramedullary nail was used to fix their fractures initially. Eleven out of twelve patients recovered following the second stage of the procedure in an average of 4 months after surgery. In our case, the fracture union was achieved two months after the second stage with no need for re-intervention afterward.

## Conclusion

The findings of this report are notable due to the fact that data about the Masquelet procedure on the upper extremity is still limited in comparison to the lower extremity reports. Moreover, the patient of this report did not experience any neurovascular complications, and the follow-up visits remained uneventful. Further research and clinical series will hopefully elucidate the grafting methods and materials necessary to optimize the healing process in patients undergoing the Masquelete procedure.

## Ethical approval

Our study is a case report without any intervention on the patient, and informed consent has been obtained for publication.

## Consent

Written informed consent was obtained from the patient for publication of this case report and accompanying images. A copy of the written consent is available for review by the Editor-in-Chief of this journal on request.

## Sources of funding

None.

## Author contribution

A.T.: idea, revising the manuscript, confirming final draft, guaranteeing all details of the project. P.S.: data collection, literature review, writing the initial draft, revising the manuscript, confirming final draft, guaranteeing all details of the project. P.M.H.A.: data collection, literature review, writing the initial draft, revising the manuscript, confirming final draft, guaranteeing all details of the project. N.G.: data collection, revising the manuscript, confirming final draft, guaranteeing all details of the project. D.H.: data collection, revising the manuscript, confirming final draft, guaranteeing all details of the project. A.N.: idea, supervision, revising the manuscript, confirming final draft, guaranteeing all details of the project.

## Conflicts of interest disclosure

The authors declare that they have no conflicts of interest.

## Guarantor

Alireza Taabbodi, Parmida Shahbazi, Payman Mohammad Hosseini Azar, Niloofar Gholami, Dorsa Hadavi, Arvin Najafi.

## Provenance and peer review

Not commissioned, externally peer-reviewed.
